# Implicit and explicit racial prejudice and stereotyping toward Black (vs. White) Americans: The prevalence and variation among genetic counselors in North America

**DOI:** 10.1002/jgc4.1648

**Published:** 2022-11-07

**Authors:** Nao Hagiwara, Conor Duffy, John Quillin

**Affiliations:** 1 Department of Psychology, Virginia Commonwealth University, Richmond, Virginia, USA; 2 Department of Pediatrics, Virginia Commonwealth University School of Medicine, Richmond, Virginia, USA

**Keywords:** Attitudes, Beliefs, Explicit bias, Genetic counselors, Implicit bias

## Abstract

Research has shown that patient experiences and outcomes of genetic counseling are not equitable across racial categories, disadvantaging Black patients relative to White patients. One major factor contributing to such racial disparities might be genetic counselor racial bias. The present study examined the prevalence of and variation in racial bias toward Black (vs. White) Americans among genetic counselors in North America. This study extends the current literature of racial disparities in experiences and outcomes of genetic counseling by distinguishing prejudice (negative feelings or attitudes) and stereotyping (beliefs) at the implicit and explicit levels as well as by including both certified genetic counselors and genetic counseling trainees. Two-hundred and fifteen genetic counselors (107 genetic counselors Board-certified by the American Board of Genetic Counseling, 108 genetic counseling trainees from Accreditation Council for Genetic Counseling accredited programs) completed four measures in a random order: the Race Implicit Association Test (IAT, for implicit prejudice), feeling thermometer (for explicit prejudice), the Medical Cooperativeness IAT (for implicit stereotyping), and a self- report measure of explicit stereotypes (for explicit stereotyping). On average, genetic counselors (both certified genetic counselors and genetic counseling trainees) were slightly to moderately in favor of White Americans over Black Americans at the implicit level. They were also slightly more likely to associate “medically cooperative” stereotypes with White Americans more than Black Americans implicitly. In contrast, genetic counselors, on average, did not display either explicit prejudice or explicit negative stereotyping, which may reflect social desirability concerns among genetic counselors. However, genetic counselors as a group strongly endorsed stereotypes related to mistrust (mistrustful of the healthcare system, skeptical of genetic testing, mistrustful of genetic counselors) to be more true for Black (vs. White) Americans. Finally, our study revealed relatively large variability in each type of bias across genetic counselors. Future research should examine how such variability in each type of bias is associated with patient experiences and outcomes of genetic counseling.

## 1 | INTRODUCTION

Racial health disparities exist in a wide range of diseases, including diseases that are addressed by genetic counselors, such as cancer, cardiovascular disease, and Alzheimer disease (National Academies of Sciences, Engineering, and Medicine, 2017). Health disparities are different from health differences that are attributable to genetic variations; they are differences in health status specifically due to social, political, economic, and psychological processes (US Department of Health and Human Services, 2008). Thus, racial health disparities refer to variations in the health status across racial categories that cannot be explained directly by heredity and disproportionately burden racial/ethnic minorities. A good example of this is pervasive and persistent racial cancer health disparities. Even though hereditary cancer is distributed similarly across racial categories (Alvarado et al., 2020), Black Americans are more likely to die from cancer than White Americans (American Cancer Society, 2019).

Because genetic counselors strive to reduce disease burden by addressing not only medical but also psychological and familial concerns related to genetic disease, genetic counselors have the potential to play a central role in reducing racial health disparities in genetic disease. However, experiences and outcomes of genetic counseling are not equitable across racial categories (Southwick et al., 2020). One major reason for such racial disparities in patient experiences and outcomes of genetic counseling might be due to racial bias among genetic counselors. Prior research on physicians provides strong evidence that physicians, on average, hold moderate levels of implicit bias against Black Americans (Sabin et al., 2009) and that physician implicit bias can negatively impact the quality of patient-provider communication, particularly during racially discordant medical interactions with Black patients (Institute of Medicine, 2003). Consistent with these findings in physicians, recent studies have shown that genetic counselors in the United States, on average, also hold moderate levels of implicit prejudice against Black Americans (Lowe et al., 2020a, 2020b; Pollock et al., 2022; Schaa et al., 2015). Studies have further shown that genetic counselor implicit prejudice is associated with racial disparities in patient- genetic counselor communication, such as affective display and emotionally responsive communication (Schaa et al., 2015) and informational individuation (Lowe et al., 2020b).

Although prior research provides initial evidence that genetic counselors’ bias contributes to racial disparities in experiences and outcomes of genetic counseling, there are methodological and theoretical limitations that must be addressed to further advance the field. Methodologically, many of these studies that examined genetic counselor bias used data that were collected from the same sample (Lowe et al., 2020a, 2020b; Schaa et al., 2015), while another study was limited to a single cohort of recent graduates (Pollock et al., 2022). Recent social psychology research provides strong evidence that an aggregate of IAT scores obtained from individual participants in a given environment (e.g., college campuses) or a geographical location (e.g., zip codes) is significantly associated with markers of systemic racism in that particular environment or location (Payne et al., 2019; Payne & Hannay, 2021; Vuletich & Payne, 2019). This implies that individuals’ IAT scores may shift with cultural, historical, and political climate within institutions. Because an increasing number of genetic counseling programs in North America have invested in diversity, inclusion, cultural competency and equity training in recent years, the levels of implicit racial prejudice in genetic counselors might have also shifted accordingly. Thus, replication of the findings in a different, broad, and more recent cross-sectional sample is essential for empirically testing the generalizability of findings from prior research. Theoretically, the previous studies assessed only implicit prejudice, which is one of the four types of bias that could potentially result in racial disparities in experiences and outcomes of genetic counseling.

Racial bias consists of prejudice (i.e., negative feelings toward racial minorities) and stereotyping (i.e., beliefs about racial minorities), both of which can operate at implicit and explicit levels. At the implicit level, feelings and beliefs are activated spontaneously and automatically; in contrast, feelings and beliefs at the explicit level are deliberate and reflect effortful processes (Wilson et al., 2000). Distinguishing these four types of bias (implicit prejudice, explicit prejudice, implicit stereotyping, and explicit stereotyping) is critical because they predict different behaviors. Specifically, social psychology research provides strong evidence that implicit bias better predicts unintentional, spontaneous behaviors than explicit bias, whereas explicit bias better predicts intentional, deliberate behaviors than implicit bias (Dovidio & Gaertner, 2010).

Consistent with these findings, different types of healthcare provider bias have been also found to be associated with different aspects of patient care (Hagiwara et al., 2020). For example, healthcare providers’ *implicit* prejudice is associated with providers’ spontaneous communication behaviors during racially discordant medical interactions, such as verbal dominance (Cooper et al., 2012). In contrast, providers’ greater *explicit* stereotyping is associated with deliberate behaviors, particularly clinical recommendations (Calabrese et al., 2018). Although there is no evidence to suggest that provider *explicit* prejudice is associated directly with providers’ deliberate behaviors, which is likely due to the professional norms that strongly condemn discriminatory practices, there is some evidence suggesting that provider explicit prejudice moderates the association between provider implicit prejudice and provider communication behaviors (Penner et al., 2010). Finally, more research has to examine how provider implicit stereotyping may manifest in providers’ behaviors during racially discordant medical interactions before we can make any conclusion.

The present study addresses those methodological and theoretical limitations by assessing all four types of bias in a new sample of genetic counselors in North America. The present study also extends the literature of genetic counselor bias by including both certified genetic counselors and genetic counseling trainees. (We will use the inclusive term “genetic counselors” when referring to both certified genetic counselors and genetic counseling trainees). Inclusion of trainees is important because they provide a significant proportion of genetic counseling; 40% of genetic counselors report “supervising students” is a significant part of their job (National Society of Genetic Counselors [NSGC], 2021). In sum, the goal of this study is to document the prevalence of racial bias toward Black Americans among certified genetic counselors and genetic counseling trainees.

Consistent with the previous studies of genetic counselors (Lowe et al., 2020a, 2020b; Pollock et al., 2022; Schaa et al., 2015), we hypothesized that genetic counselors would display implicit prejudice toward Black (versus White) Americans. Because no other study to-date, to our knowledge, has assessed the remaining three types of bias (explicit prejudice, implicit stereotyping, explicit stereotyping) toward Black Americans in genetic counselors, we formulated our predictions by drawing on findings from prior research of physician bias.

Findings on the presence/absence of explicit prejudice in physicians have been mixed. On the one hand, a study that analyzed data collected from a large sample of physicians (over 2500 physicians) via the Project Implicit demonstration website has documented that physicians in the United States, on the national average, reported a slight preference for White Americans relative to Black Americans. On the contrary, individual studies with smaller samples of physicians generally find no explicit prejudice against Black Americans (Green et al., 2007) or even a slight preference for Black Americans (Guedj et al., 2021), which is likely to reflect overcompensation due to motivation to appear non-prejudiced (Dunton & Fazio, 1997). One major reason for these divergent findings is likely due to the Project Implicit website’s ability to eliminate social desirability bias. We believe that the Project Implicit website increases a sense of anonymity and minimizes social desirability concerns because anyone across the globe can voluntarily take their demonstration IATs without providing any identifiable information. Consequently, respondents would feel less pressure to regulate their responses to measures of explicit prejudice. Because we tried to minimize social desirability bias in the current study (e.g., voluntary participation through self-enrollment, use of organizational listservs, separating the main survey and participant payment information), we hypothesized that genetic counselors would display explicit prejudice against Black Americans.

We also predicted that genetic counselors would display implicit stereotyping (particularly associating “medical uncooperativeness” with Black, relative to White, Americans) by drawing on prior research that has consistently shown physicians perceive Black Americans to be less medically cooperative than White Americans at the implicit level (Green et al., 2007; Sabin & Greenwald, 2012). Finally, we hypothesized that genetic counselors would display explicit negative stereotyping. Research has shown that physicians hold explicit negative stereotypes about Black Americans, such as unintelligent, noncompliant, and sexually promiscuous (Calabrese et al., 2014; Hoffman et al., 2016). Recent research also suggests physicians hold explicit stereotypes about Black Americans, such as beliefs about mistrust, cost concerns, and psychological distress, that may serve as barriers for genetic counseling referral (Ademuyiwa et al., 2021). Finally, there is some evidence showing that reports of stereotypes are associated with implicit prejudice (Byrd et al., 2015).

## 2 | METHODS

### 2.1 | Participants

To recruit certified genetic counselors, we e-blasted a study invitation with a link to an online study to potentially eligible individuals through the American Board of Genetic Counseling (ABGC). To reach genetic counseling trainees from programs that are accredited by the Accreditation Council for Genetic Counseling (ACGC), we emailed members of the Association of Genetic Counseling Program Directors through their group email address, requesting that they share the study invitation with their students. Interested individuals were first asked to complete the screening. Only individuals who met the eligibility criteria (i.e., either being certified by the ABGC or currently being in an ACGC-accredited training program in the United States) were able to self-enroll into the study. A total of 412 participants self-enrolled in the study. However, 197 participants were excluded from the analysis because they did not complete all four measures of bias (188 missing all four measures, 2 missing three measures, 4 missing two measures, and 3 missing one measure), resulting in 215 analyzable cases (107 certified genetic counselors and 108 genetic counseling trainees).

### 2.2 | Instrumentation

#### 2.2.1 | Implicit racial prejudice

Implicit prejudice was assessed with Race Implicit Association Test (IAT; Greenwald et al., 1998). In the Race IAT, 12 photographs of faces and 16 words (marvelous, superb, pleasure, beautiful, joyful, glorious, lovely, wonderful, tragic, horrible, agony, painful, terrible, awful, humiliate, and nasty) are presented one at a time. Participants are instructed to categorize items into four categories: two racial groups (Black vs. White) and two evaluations (good vs. bad), which are presented in pairs. The premise is that participants would respond more quickly when the racial group and valence mapped onto the same response are strongly associated than when they are weakly associated. The IAT is scored by computing *D* score that ranges from −2.0 to 2.0 (Greenwald et al., 2003), with greater positive values indicating implicit preference for White Americans over Black Americans and greater negative values indicating implicit preference for Black Americans over White Americans. A value of zero indicates no implicit preference between Black and White Americans. Generally, *D* scores are interpreted as “slightly biased” (±0.15), “moderately biased” (±0.35), and “strongly biased” (±0.65; Epifania et al., 2020). The IAT is reliable (*α*s between 0.70 and 0.90; Hofmann et al., 2005) and has been well-validated (Nosek et al., 2005).

#### 2.2.2 | Explicit racial prejudice

Explicit racial prejudice was assessed with a widely used, well-validated “feeling thermometer” (Campbell, 1971), in which participants rate their feelings toward certain racial groups using a scale ranging from 0 (very cold) to 100 (very warm). We created composite explicit prejudice scores, which mirror *D* scores in the IATs, by subtracting a score for Black Americans from a score for White Americans. Therefore, greater positive values indicate explicit preference for White Americans over Black Americans while greater negative values indicate explicit preference for Black Americans over White Americans. A value of zero indicates no explicit preference between Black and White Americans.

#### 2.2.3 | Implicit racial stereotyping

Implicit racial stereotyping was assessed with the medical cooperativeness IAT, which is the most commonly used implicit measure of racial stereotyping in racial health disparities research (Green et al., 2007; Sabin & Greenwald, 2012). The design of the medical cooperativeness IAT is similar to the one of the Race IAT. Instead of 16 words that were to be categorized into evaluations (good vs. bad), participants were presented with 12 words that were to be categorized into stereotypes (medically cooperative vs. uncooperative). Those 12 words were: willing, cooperative, compliant, reliable, adherent, helpful, reluctant, doubting, hesitant, apathetic, resistant, and lax. *D* scores were computed using the same algorithm as for the Race IAT; thus, greater positive values indicate implicit association of White (vs. Black) Americans with medical cooperativeness, while greater negative values indicate implicit association of Black (vs. White) Americans with medical cooperativeness.

#### 2.2.4 | Explicit racial stereotyping

Explicit racial stereotyping was assessed with two new measures that were created for this study: a 32-item measure designed to assess general racial stereotypes (e.g., unintelligent, lazy, and low socioeconomic status), and a 34-item measure designed to assess racial stereotypes specifically in the medical context (e.g., medical compliance, health literacy, and motivation). All items that were included in the general racial stereotyping measure were adapted from the study by Wittenbrink et al. (1997). Twelve items included in the medical racial stereotyping measure were adapted from the study by Hoffman et al. (2016), while the remaining 22 items were created by the authors. In both measures, participants were instructed to read statements and indicate the extent to which each statement describes characteristics of Black vs. White Americans, using a Likert scale that ranges from 1 (very untrue) to 6 (very true). To be consistent with the other three measures of bias, we computed composite scores that compare Black vs. White Americans. More specifically, we reverse-coded negatively worded items and computed a composite score for general stereotypes and medicine-specific stereotypes separately by subtracting the average score for Black Americans from the average score for White Americans. Greater positive values indicate explicit association of White Americans (vs. Black Americans) to more positive stereotypes, while greater negative values indicate explicit association of Black Americans (vs. White Americans) to more positive stereotypes.

### 2.3 | Procedures

Participants completed the demographic information (which was administered on Qualtrics) and then proceeded to complete all four measures of bias (which were administered using Inquisit 6 Web). The presentation order of the four measures was randomized across participants. Additionally, the presentation order of items was randomized within each measure. Upon completion of the entire study, participants were directed to another survey administered on Qualtrics and given an opportunity to provide their names and email addresses to receive a $25 eGift card for their participation. Data were collected between August 18, 2021, and November 23, 2021. The study was reviewed and approved by the Virginia Commonwealth University Institutional Review Board (HM20022251) as human subject research.

### 2.4 | Data analysis

Data were analyzed using SPSS Statistics 27.0. The primary analysis aimed to document means, standard deviations, ranges, and distributions of the four types of bias. We also examined whether group means are significantly different between certified genetic counselors and genetic counseling trainees by conducting independent-samples *t*-tests. The secondary analysis aimed to document the presence/absence of each type of bias by conducting a one-sample *t*-test with score 0 as the reference point for each type of bias. The tertiary analysis aimed to explore the associations between participant demographic characteristics and four types of biases. First, we computed bivariate correlations between all demographic characteristics (i.e., age, race/ethnicity, sex/gender, professional status, and year in practice/training) and the four types of bias. Second, we proposed to follow-up with a stepwise regression with the backward elimination for each composite score as an outcome. Each model would have started with all demographic characteristics, and we would delete one characteristic at a time until no variable could be removed without losing a model fit (*ΔR*^*2*^, *p* < 0.05). Categorical variables (i.e., professional status, gender, race/ethnicity, and years in training [only for trainees]) were dummy-coded, and continuous variables (e.g., age and years in practice [only for certified genetic counselors]) were grand-mean-centered before being entered into the model. We used *α* = 0.05 as the cut-off criterion for the secondary and tertiary analyses. The analysis plan was pre-registered on the Open Science Framework website (https://osf.io/7s5ma).

In addition to the main analyses, we conducted ancillary analysis of explicit racial stereotyping. The composite scores for explicit racial stereotyping computed solely based on the valence of stereotypes (positive vs. negative) can mask potential variations in the stereotype content (e.g., some may perceive Black Americans to be poorer, but not lazier, than White Americans). To explore how participants responded to each specific stereotype, we also computed a difference score for each item (subtracting a score for Black Americans from a score for White Americans on a given item, so higher values indicated greater endorsement of a particular stereotype as being true for Whites, relative to Blacks). Specifically, we first examined the means, standard deviations, and ranges to assess the overall level and variability of specific explicit stereotypes among genetic counselors. We also assessed the prevalence of specific explicit stereotypes by examining the percentage of genetic counselors who endorse a given stereotype as more true for Black Americans, White Americans, or neither group.

## 3 | RESULTS

Participant characteristics are presented in Table 1. Although genetic counseling trainees are more diverse than certified genetic counselors in terms of both race/ethnicity and sex/gender, which is consistent with the recent effort in the field of genetic counseling to promote diversity (Channaoui et al., 2020), the overwhelming majority of participants in the current sample were still White and cis- women. Thus, race/ethnicity and sex/gender were dichotomized, with White and cis woman as the reference groups.

### 3.1 | The prevalence and distribution of the four types of bias

**FIGURE 1:**
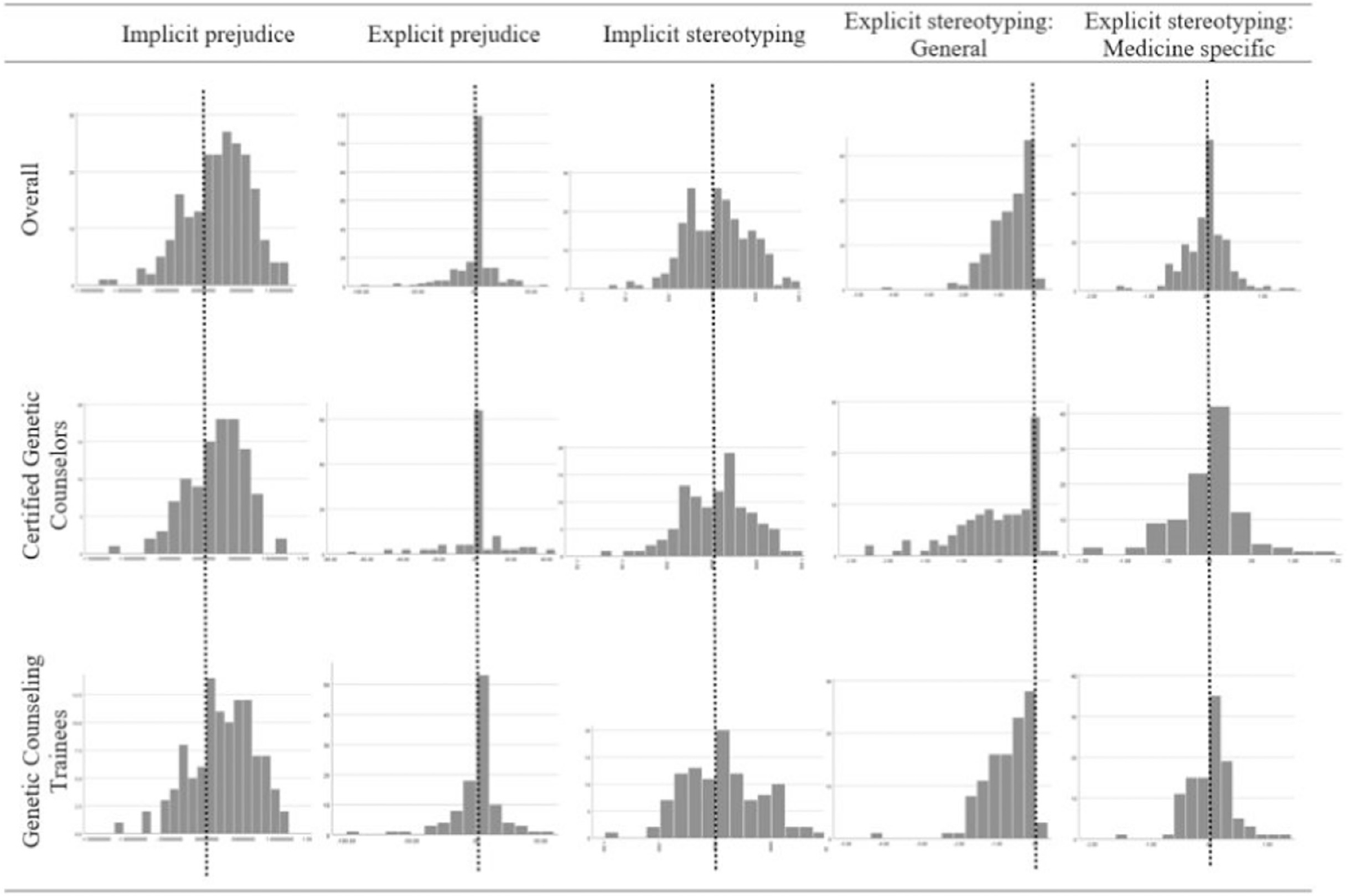
Distribution of Each of the Four Types of Bias among Genetic Counselors. In each bar graph, the x-axis indicates scores, and the y-axis indicates the frequency. The dotted lines indicate the means and were used to align three graphics (overall, certified genetic counselors only, genetic counseling trainees only) within each type of bias for a comparison.

Table 2 summarizes means, standard deviations, and ranges of each of the four types of bias for the entire sample as well as for certified genetic counselors and genetic counseling trainees separately. We also visually inspected the distribution of each type of bias among the entire sample, certified genetic counselors only, and genetic counseling trainees only (Figure 1). Means of implicit prejudice and implicit stereotyping were greater than zero, whereas those of explicit prejudice and explicit stereotyping were less than zero. That is, a trend was for genetic counselors to display pro- White/anti-Black bias at the implicit level but pro-Black/anti-White bias at the explicit level on average.

As reflected in standard deviations, ranges, and score distributions, there was relatively large variability in each type of bias. That is, some genetic counselors displayed a given type of bias more than other genetic counselors. The visual inspection of figures further suggests that score distributions of explicit prejudice and explicit stereotyping (particularly general stereotypes) were highly skewed and leptokurtic, while implicit prejudice and implicit stereotyping were distributed normally. To test our observation statistically, we followed up with examinations of skewness and kurtosis as well as the Kolmogorov–Smirnov normality tests (Kim, 2013) for each type of bias, which are presented in Table 3. Consistent with our observation, skewness and kurtosis were high for explicit prejudice and explicit general stereotypes. Although skewness was low for explicit medicine-specific stereotyping, kurtosis was still high. The Kolmogorov–Smirnov normality tests confirmed that only implicit prejudice and implicit stereotyping were normally distributed.

Finally, independent-samples *t*-tests revealed that certified genetic counselors and genetic counseling trainees, on average, did not differ in any of the four types of bias. The distributions of the four types of bias were also all similar between certified genetic counselors and genetic counseling trainees. Therefore, the subsequent analyses were conducted with the overall sample.

### 3.2 | The presence/absence of the four types of bias

Consistent with our prediction, genetic counselors displayed implicit prejudice that is significantly higher than zero, *t*(214) = 6.96, *p* < 0.001, *d* = 0.47. This indicates they were slightly to moderately in favor of White Americans over Black Americans at the implicit level. Genetic counselors also displayed implicit stereotyping that is significantly higher than zero, *t*(214) = 2.11, *p* < 0.036, *d* = 0.37. They were slightly more likely to associate White (vs. Black) Americans with “medically cooperative” stereotypes at the implicit level.

Contrary to our predictions (and prior research on physicians), genetic counselors, on average, did not display either explicit prejudice, *t*(214) = −0.98, *p* = 0.328, or explicit stereotyping specific to the context of medicine, *t*(214) = −0.09, *p* = 0.927. Furthermore, genetic counselors displayed explicit general stereotyping that is opposed from what we expected, *t*(214) = −15.55, *p* < 0.001, *d* = 0.60. Specifically, they were more likely to associate Black (vs. White) Americans with positive general characteristics.

### 3.3 | Demographic characteristics associated with the four types of bias

Table 4 presents bivariate correlations among all variables. Examinations of Point Biserial coefficients suggest that only participant race/ethnicity was associated with some of the biases, specifically explicit prejudice (*r* = −0.222, *p* = 0.001) and explicit stereotyping specific to the medical context (*r* = 0.162, *p* = 0.018). These coefficients suggest that, compared with White genetic counselors, non-White genetic counselors were *less* likely to display explicit racial prejudice toward Black Americans but more likely to associate Black Americans with negative medical stereotypes. Because none of the other demographic characteristics were associated with any of the biases, we did not conduct backward regressions.

### 3.4 | Ancillary analyses of explicit racial stereotyping

Table S1 in Supporting Information presents means, standard deviations, and ranges of each explicit stereotype for the entire sample, certified genetic counselors only, and genetic counseling trainees only. Positive numbers indicate that genetic counselors, on average, endorse a given stereotype to be more true for White Americans relative to Black Americans. In contrast, negative numbers indicate that genetic counselors, on average, endorse a given stereotype to be more true for Black Americans relative to White Americans.

The pattern of the endorsement of explicit stereotypes was similar between certified genetic counselors and genetic counseling trainees in general, except for four stereotypes (humorous, cheerful, sheltered, and worried about privacy of genetic test results). Specifically, the degree of the endorsement of “humorous” and “cheerful” for Black (relative to White) Americans was greater for genetic counseling trainees, on average, than for certified genetic counselors. In contrast, the degree of the endorsement of “sheltered” for White (relative to Black) Americans was greater for certified genetic counselors than for genetic counseling trainees. Finally, certified genetic counselors endorsed “worried about privacy of genetic test results” to be more true for White Americans, whereas genetic counseling trainees endorse it to be more true for Black Americans. However, these differences should be interpreted with caution given an increased chance of making Type I errors due to multiple testing. In fact, when we adjusted the *p*-value using the Bonferroni correction (*p* < 0.0015), the differences between genetic counselors and genetic counseling trainees became non-significant.

One-sample *t*-tests revealed that genetic counselors overall endorsed all positive general stereotypes to be more true for Black (vs. White) Americans and all negative general stereotypes to be more true for White (vs. Black) Americans. In contrast, the pattern of the endorsement of medicine-specific stereotypes was more complicated. First, there were a number of medicine-specific stereotypes that were endorsed to be true for neither Black nor White Americans (having a sensitive sense of smell, tolerating pain well, having high levels of health literacy, worrying about privacy of genetic test results, being knowledgeable about family health history, being unable to afford genetic counseling services financially). Second, inconsistent with the results of implicit stereotyping of medical cooperativeness, genetic counselors endorsed stereotypes related to medical cooperativeness (being medically compliant, motivated to improve health, adhering to clinical recommendations, being medically cooperative) to be more true for Black (vs. White) Americans at the explicit level. Third, genetic counselors strongly endorsed stereotypes related to mistrust (mistrustful of the healthcare system, skeptical of genetic testing, mistrustful of genetic counselors) to be more true for Black (vs. White) Americans.

In addition to the average level of endorsement, we also examined the distribution of the endorsement of each specific stereotype (Table S2 in supplementary information). For this analysis, we divided genetic counselors into three groups: those who endorsed a particular stereotype to be more true for Black (vs. White) Americans (difference scores < 0), those who perceived a particular stereotype to be more true for White (vs. Black) Americans (difference scores > 0) and those who perceived a particular stereotype to be more/less true for neither Black nor White Americans (difference scores = 0). Twenty-five of the 33 explicit stereotypes were not endorsed by the majority of genetic counselors in either way. The analyses of the remaining eight explicit stereotypes revealed a clear pattern. All six stereotypes that were endorsed to be more true for White Americans than for Black Americans by the majority of genetic counselors were general negative stereotypes (i.e., exploitative, materialistic, sheltered, lazy, violent, and complaining). The two stereotypes that were endorsed to be more true for Black Americans than for White Americans by the majority of genetic counselors were both about mistrust (i.e., mistrustful of the healthcare system, mistrustful of genetic counselors).

## 4 | DISCUSSION

We assessed four types of bias (implicit prejudice, explicit prejudice, implicit stereotyping, and explicit stereotyping) in a sample of 215 genetic counselors, including both genetic counselors board-certified by the ABGC and genetic counseling trainees in ACGC accredited programs. Based on the previous studies that examined the role of genetic counselors in patient care (Lowe et al., 2020a, 2020b; Schaa et al., 2015), we predicted that genetic counselors will display implicit prejudice toward Black (versus White) Americans. We also hypothesized that genetic counselors will display explicit prejudice, implicit stereotyping, and explicit stereotyping toward Black Americans based on prior research documenting the presence of explicit prejudice (Sabin et al., 2009), implicit stereotyping (Green et al., 2007; Sabin & Greenwald, 2012), and explicit stereotyping (Calabrese et al., 2014; Hoffman et al., 2016) in physicians. Our hypotheses were partially supported.

Consistent with our predictions, genetic counselors displayed both implicit prejudice and implicit stereotyping overall. However, the levels of these biases were somewhat lower than those reported in prior research. More specifically, the level of implicit prejudice reported in the current sample of genetic counselors is considered “small-to-moderate” based on the conventional rule (Epifania et al., 2020), whereas the level reported in the previous three studies of implicit prejudice among genetic counselors was in the range of “moderate-to-strong” (*M* = 0.42). This might be due to at least two reasons. First, participants who self-enrolled in the current study and were willing to complete all four measures of racial bias may be less racially biased than the general genetic counselor population. Second, there might be the time effect. Implicit prejudice data for all genetic counselors (not just recent graduates) used in the previous three studies were collected in 2010 (Lowe et al., 2020a, 2020b; Schaa et al., 2015), more than a decade ago. Recent research has shown, using data collected on the Project Implicit demonstration website, that the national average of the race IAT scores has decreased from 0.33 in 2007 down to 0.30 in 2016 (Charlesworth & Banaji, 2019).

Turning to the level of implicit stereotyping, the average medical cooperativeness IAT scores in the previous studies ranged from anywhere between 0.22 (Green et al., 2007) and 0.25 (Sabin & Greenwald, 2012) in physicians, whereas the average score of genetic counselors in the current sample was 0.05. Given that this was the first study to assess implicit racial stereotyping in genetic counselors, replication studies are encouraged. However, one potential explanation for the difference in the levels of implicit racial stereotyping between prior research on physicians and the current study on genetic counselors might be training. As they undergo medical training, physicians are encouraged to pay attention to patient race when they make decisions about treatment options, which is reflected in the inclusion of race in clinical algorithms and textbooks (Vyas et al., 2020). The erroneous assumption here is that physicians could better make inferences about a particular patient by simply knowing patient race and what is “typical” (or more accurately “stereotypical”) of that race. In other words, physicians are trained to use racial stereotypes in their practice (Satel, 2002). On the other hand, genetic counselors, especially recently, are increasingly trained to apply their practice without differentiating by race. For example, historically race/ethnicity dictated what type of carrier screening genetics providers, including genetic counselors, offer patients (e.g., Sickle Cell carrier screening for Black patients). Currently, however, organizations like the American College of Medical Genetics and Genomics recommend: “All individuals, regardless of race or ethnicity, are offered screening for the same set of conditions” (Nazareth et al., 2015). This sentiment has extended to the conduct and reporting of race in genetics- related research (Brothers et al., 2021).

Contrary to our predictions, participating genetic counselors, on average, did not display either explicit prejudice or explicit negative stereotyping toward Black Americans. The lack of evidence supporting the presence of explicit prejudice among genetic counselors might be also due to the selection bias described above. Alternatively, we might have failed to minimize participants’ social desirability concerns to the same extent as the Project Implicit website. Before being self-enrolled into the study, participants were informed that the study aimed to “assess racial attitudes and beliefs at the implicit and explicit levels among genetic counseling providers (both certified genetic counselors and genetic counseling trainees) across the nation.” This prompt might have made their professional membership salient and increased their desire to present their profession in a positive light. Participants were also told that they would be given an opportunity to provide their contact information to receive participant payments at the end of the study in a separate survey, which might have increased self- awareness before they started the study. Social psychology research has shown self- awareness (even just looking at oneself in a mirror) can increase behavioral responses consistent with one’s personal moral standards (Gibbons, 1990).

Finally, participating genetic counselors, on average, did not display overall explicit negative stereotypes about Black Americans. Examinations of individual stereotypes also confirmed that many individual stereotypes were not endorsed by the majority of genetic counselors. However, a clear pattern emerged among the stereotypes that were endorsed. Specifically, the majority (ranging from 50.7% to 66.1%) of genetic counselors endorsed six negative general stereotypes (i.e., exploitative, materialistic, sheltered, lazy, violent, and complaining) to be more true for White Americans than for Black Americans and two mistrust-related stereotypes specifically in the context of medicine (i.e., mistrustful of the healthcare system, mistrustful of genetic counselors) to be more true for Black Americans than for White Americans.

One major difference between those two categories of stereotypes is valence that signals inherent good or bad. The first set of six stereotypes are clearly about negative personal characteristics and reporting that they endorse those negative characteristics to be true for Black Americans more than White Americans can risk appearing prejudiced. In contrast, reporting that they endorse those negative characteristics to be true for White Americans more than Black Americans could potentially signal individuals’ egalitarian values or even knowledge/awareness of systemic racism that produces and reinforces negative stereotypes about Black Americans. Thus, the results that six (out of the eight) general negative stereotypes were endorsed by the majority of genetic counselors might reflect overcompensation (Dunton & Fazio, 1997). In fact, the distributions of the remaining 10 stereotypes that were not endorsed by the majority of genetic counselors also support the idea of overcompensation. Specifically, we compared the percentages of genetic counselors endorsing specific stereotypes to be more true for either Black or White Americans (i.e., excluding those who did not endorse the stereotypes from the comparisons). When stereotypes were positive (intelligent, educated, industrious, responsible, playful, humorous, cheerful, and athletic), there were always more genetic counselors who endorsed those stereotypes to be more true for Black Americans. However, when stereotypes were negative (stubborn and poor), there were always more genetic counselors who endorse those stereotypes to be more true for White Americans.

In contrast to the first set of six general stereotypes with clear valence that signals inherent bad, the second set of two medicine-specific stereotypes tended to be valence-neutral or -ambiguous. Mistrust is often induced by actions (or inactions) by other people or organizations, placing a focus on external factors (Benkert et al., 2019). So, it does not signal that a person is inherently good or bad to mistrust something. Consequently, participants’ social desirability concern might have been reduced for the medicine-specific, mistrust-related stereotypes, enabling them to report their perceptions without deliberate modifications (Crandall & Eshleman, 2003). Supporting the association between valence of stereotypes and overcompensation, for medicine-specific stereotypes that may signal inherent good (e.g., medically compliant, motivated to improve one’s health, and medically cooperative), there were more genetic counselors who endorsed them as being more true for Black Americans than for White Americans, although the majority of genetic counselors still did not endorse these stereotypes to be more/less true for either group. One exception to this pattern was the attribute “having high levels of health literacy.” This stereotype might have been different from other medicine-specific stereotypes that may signal inherent good in that it places focus more on structural factors (e.g., education and income) rather than individuals per se. In contrast, for a medicine-specific stereotype that may signal inherent bad (i.e., do not want to know about future health risks), there were more genetic counselors who endorsed them as being more true for White Americans than for Black Americans. We acknowledge that there has been an ongoing discussion surrounding ethical issues of the “Right to Know/Not to Know” (Berkman & Hull, 2014) and that the attribute “don’t want to know about future health risks” should not (or cannot) be characterized as either bad or good in some medical contexts, such as pregnancy or late-onset diseases without known prevention (e.g., Huntington disease). However, much of cancer genetic counseling involves providing knowledge that can significantly reduce morbidity and mortality for patients and their family members. In such cases, traditional arguments for a right not to know may not apply, or at least should be reconsidered.

It should be noted that the average levels of the four types of bias did not differ significantly between certified genetic counselors and genetic counseling trainees. This may imply that genetic counseling training may not have been effective in reducing genetic counselor bias despite its effort to promote cultural competency (Benkert et al., 2019; Pollock et al., 2022). Finally, the relatively large variability that we documented in all four types of bias should be noted. Documenting variability in each type of bias is as important as assessing the overall levels of bias in genetic counselors. It is the variability in bias across genetic counselors (not the average levels of bias) that informs why some genetic counselors treat Black and White patients differently while other genetic counselors treat Black and White patients in the same ways. In fact, the variability in implicit racial prejudice that we documented in our overall sample of genetic counselors (*SD* = 0.43) was compatible to that reported in two previous samples of genetic counselors (*SD*s = 0.36–0 .40; Lowe et al., 2020a, 2020b; Pollock et al., 2022; Schaa et al., 2015) as well as in three samples of physicians (*SD*s = 0.40–0.47; Green et al., 2007; Sabin & Greenwald, 2012; Sabin et al., 2009). Likewise, the variability in implicit racial stereotyping in our overall sample of genetic counselors (*SD* = 0.37) was similar to that in two samples of physicians (*SD*s = 0.39–0.42; Green et al., 2007; Sabin & Greenwald, 2012).

### 4.1 | Study limitations and future research directions

As stated earlier, one potential methodological limitation that might have impacted the current results might be selection bias. Before self-enrolling into the study, genetic counselors were made aware that the goal of the study was to assess racial attitudes and beliefs at the implicit and explicit levels. The topic can be uncomfortable to some, discouraging them from participating in the study voluntarily. In fact, 188 participants who self-enrolled into the study decided to withdraw from the study before completing any measures of bias. Consequently, our results might have been skewed to reflect the levels of bias among genetic counselors who are comfortable with sharing their racial attitudes and beliefs and who may be less racially biased than the general genetic counselor population.

Another limitation is that we had to collapse racial/ethnic categories into only two groups (White and non-White) because there were too few racial/ethnic minority genetic counselors. Prior research has shown that the levels of implicit prejudice and explicit prejudice toward Black Americans differ across respondent race/ethnicity (Sabin et al., 2009). Thus, categorizing all racial/ethnic minority genetic counselors into a single group can mask meaningful differences in the prevalence of and variability in the four types of bias. However, this limitation actually reflects a larger, persistent issue in the field of genetic counseling: the lack of diversity. According to a report published by the National Society of Genetic Counselors in 2021, between 2016 and 2020, 91.5% of genetic counselors self- identified as White (NSGC, 2021).

Third, although we suspect that genetic counselors’ social desirability bias might have played a role in explaining the absence of explicit prejudice or explicit stereotyping (and the pattern of the results that are consistent with the process of overcompensation), we were unable to test the idea empirically. In the context of the current study that focuses on racial prejudice and stereotyping, social desirability concerns are likely to be associated with motivation to respond without prejudice. That is, individuals who are highly motivated to respond without prejudice are more likely than those with low motivation to modify their responses. Future research may utilize well-validated measures like the Motivation to Control Prejudice Reactions Scale (Dunton & Fazio, 1997) and the Internal Motivation to Respond without Prejudice Scale and External Motivation to Respond without Prejudice Scale (Plant & Devine, 1998).

Fourth, we did not directly ask practicing genetic counselors to indicate whether they were practicing in the United States at the time of data collection. However, because all of the practicing genetic counselors in the current study were board-certified by the ABGC and also recruited through the ABGC’s e-blast, they likely were US-based at the time of enrollment. Additionally, research has shown that implicit preference for White Americans to Black Americans has been reported consistently across 34 countries (*M* = 0.38, *SD* = 0.41, ranged from 0.27 in Serbia to 0.49 in Japan; Charlesworth et al., 2022), which is comparative to the national average in the United States. (*M* = 0.32, *SD* = 0.44; Charlesworth & Banaji, 2019). This implies that residing country at the time of data collection might have had little impact on the overall levels of implicit racial prejudice in the current sample of practicing genetic counselors.

Finally, the present study was exploratory in nature, and its goal was to document the prevalence of and variability in the four types of bias among genetic counselors. Thus, findings from the present study do not directly speak to their consequences for patient care or contributions to racial disparities in patient experiences and outcomes of genetic counseling, and thus ultimately in racial health disparities. An important next research step is to examine whether and how each type of genetic counselor bias manifests in what aspects of care (e.g., patient-genetic counselor communication, clinical discussion topics, and clinical recommendations).

### 4.2 | Practical implications

In contrast to a large literature on physician bias and its role in racial healthcare disparities, research on the role of genetic counselor bias in the genetic counseling process has been limited. This is despite the fact that there has been a rapidly increasing discourse about and recognition of racial disparities in the genetic counseling process (Southwick et al., 2020). Genetic counselor bias may play a particularly important role in explaining the racial disparities in patient experiences and outcomes of genetic counseling because genetic counseling encounters for Black patients are likely to be racially discordant due to the lack of diversity in the field as discussed earlier. Only 1.4% of genetic counselors self-identify as Black/African American (NSGC, 2021), while 12.4% of the US population are Black/African American (U.S. Census Bureau, 2020). Because the overwhelming majority of genetic counselors (91.5%) self- identify as White (NSGC, 2021) and White genetic counselors, on average, hold implicit prejudice toward and implicit stereotyping of Black Americans, Black patients are likely to interact with White genetic counselors with implicit bias toward them when they seek genetic counseling services. Future research should examine empirically how each of the four types of bias among genetic counselors is associated with different aspects of genetic counseling processes and identify mechanisms underlying such associations in order to reduce racial disparities in patient experiences and outcomes of genetic counseling.

## 5 | CONCLUSIONS

Although there is potential for genetic counselors to play a central role in reducing racial health disparities, the benefits of genetic counseling are not equitable across racial categories (Southwick et al., 2020). Prior research on physician bias and racial healthcare disparities (Institute of Medicine, 2003) suggests that one major reason for the racial disparities in patient experiences and outcomes of genetic counseling might be due to racial bias among genetic counselors. Although recent studies have shown that genetic counselors, on average, hold implicit prejudice against Black Americans (Lowe et al., 2020a, 2020b; Schaa et al., 2015), which is predictive of racial disparities in patient- genetic counselor communication, such as affective display and emotionally responsive communication (Schaa et al., 2015) and informational individuation (Lowe et al., 2020b), implicit prejudice is just one of the four types of bias that predict different types of behaviors (Hagiwara et al., 2020). Findings from this study suggest that both certified genetic counselors and genetic counseling trainees hold similar levels of implicit prejudice toward Black Americans as well as implicit stereotyping that associate “medical cooperativeness” more to White (relative to Black) Americans and “medical uncooperative” more to Black (relative to White) Americans. Although genetic counselors, on average, did not report explicit prejudice toward or explicit negative stereotyping of Black Americans, the patterns of the results of specific explicit stereotypes suggest that the absence of these types of bias may reflect social desirability concerns among genetic counselors. Future research is encouraged to examine how the four types of bias in genetic counselors are associated with different aspects of genetic counseling processes.

## Supplementary Material

Tables S1 and S2

## Figures and Tables

**TABLE 1:** Participant characteristics

	Certified genetic counselors (*n* = 107)	Genetic counseling trainees (*n* = 108)
Age	*M* = 33.59 (*SD* = 8.09)	*M* = 25.30 (*SD* = 3.80)
Race/ethnicity		
Asian American	6 (5.6%)	11 (10.2%)
Black/African American	0	2 (1.9%)
LatinX/o/a American	2 (1.9%)	8 (7.4%)
White American	91 (85.0%)	82 (75.9%)
Multiracial	5 (4.7%)	4 (3.7%)
Not listed above	3 (2.8%)	1 (0.9%)
Sex/gender		
Cis woman	104 (97.2%)	97 (89.8%)
Cis man	3 (2.8%)	8 (7.4%)
Genderqueer/non-binary	0	2 (1.9%)
Not listed above	0	1 (0.9%)
Years in practice	*M* = 4.68 (*SD* = 4.87)	
Year in training		
1st		49 (45.4%)
2nd		59 (54.6%)

**TABLE 2:** Means, standard deviations, and range of the four types of bias among genetic counselors

			Certified genetic counselors-genetic counseling trainees comparison		
	*M (SD)*	Range	*t*	*p*	95% CI
Implicit prejudice		(−2.0 to +2.0)			
Overall	0.20** (0.43)	−1.28 to 1.08			
Certified genetic counselors	0.18 (0.41)	−1.28 to 1.07			
Genetic counseling trainees	0.22 (0.45)	−1.15 to 1.06	−0.77	0.44	[-0.16, 0.07]
Explicit prejudice		(−100 to +100)			
Overall	−1.18 (17.58)	−100.0 to 58.0			
Certified genetic counselors	−0.34 (15.95)	−68.0 to 41.0			
Genetic counseling trainees	−2.01 (19.10)	−100.0 to 58.0	0.70	0.49	[−3.06, 6.41]
Implicit stereotyping		(−2.0 to +2.0)			
Overall	0.05* (0.37)	−1.14 to 0.91			
Certified genetic counselors	0.04 (0.39)	−1.14 to 0.91			
Genetic counseling trainees	0.06 (0.35)	−0.97 to 0.91	−0.35	0.73	[−0.12, 0.08]
Explicit stereotyping: General		(−5.0 to +5.0)			
Overall	−0.64** (0.37)	−4.19 to 0.25			
Certified genetic counselors	−0.56 (0.55)	−2.25 to 0.25			
Genetic counseling trainees	−0.71 (0.64)	−4.19 to 0.06	1.86	0.06	[−0.01, 0.31]
Explicit stereotyping: Medicine specific		(−5.0 to +5.0)			
Overall	−0.003 (0.39)	−1.53 to 1.47			
Certified genetic counselors	−0.03 (0.41)	−1.47 to 1.47			
Genetic counseling trainees	0.02 (0.37)	−1.53 to 1.29	−1.01	0.31	[−0.16, 0.05]

Note:

* indicates a significant one-sample *t*−test (the secondary analysis) at *p* < 0.05 and

** at *p* < 0.01. *t*−tests presented within the table are independent sample *t*−tests, comparing certified genetic counselors and genetic counseling trainees.

**TABLE 3:** Descriptive statistics on score distributions of each type of bias

	Skewness	Kurtosis	K-S sig.
Implicit prejudice			
Overall	−0.50	0.29	0.07
Certified genetic counselors	−0.65	0.70	0.02
Genetic counseling trainees	−0.42	−0.001	0.20
Explicit prejudice			
Overall	−1.34	7.13	<0.001
Certified genetic counselors	−0.96	4.48	<0.001
Genetic counseling trainees	−1.51	8.10	<0.001
Implicit stereotyping			
Overall	−0.16	0.01	0.20
Certified genetic counselors	−0.033	0.12	0.20
Genetic counseling trainees	0.09	−0.23	0.20
Explicit stereotyping: General			
Overall	−1.47	4.76	<0.001
Certified genetic counselors	−0.98	0.58	<0.001
Genetic counseling trainees	−1.74	6.61	<0.001
Explicit stereotyping: Medicine specific			
Overall	−0.18	2.90	<0.001
Certified genetic counselors	−0.19	2.70	<0.001
Genetic counseling trainees	−0.13	3.30	0.02

*Note*: K-S sig. stands for the Kolmogorov–Smirnov normality test. Values smaller than 0.05 indicate that a distribution is significantly different from the normal distribution.

**TABLE 4:** Correlation among all variables

	1	2	3	4	5	6	7	8	9	10
1. Age	-									
2. Race/ethnicity	−0.001	-								
3.Sex/gender	0.15*	0.11	-							
4. Professional status	−0.55**	N/A	N/A	-						
5. Years in practice (certified genetic counselors only)	0.63**	−0.03	−0.05	N/A	-					
6. Year in training (genetic counseling trainees only)	−0.02	−0.01	−0.001	N/A	N/A	-				
7. Implicit prejudice	0.003	−0.004	0.09	0.05	0.04	0.01	-			
8. Explicit prejudice	−0.05	−0.22**	−0.06	−0.05	−0.06	0.07	0.15*	-		
9. Implicit stereotyping	−0.001	0.05	0.02	0.02	−0.01	0.10	0.51***	0.22***	-	
10. Explicit stereotyping: General	0.06	0.02	0.09	−0.13	0.04	0.18	0.10	0.15*	0.09	-
11. Explicit stereotyping: Medicine specific	−0.06	0.16*	0.06	0.07	0.04	−0.12	0.11	0.05	0.14*	0.17*

Note:

* indicates *p* < 0.05,

** indicates *p* < 0.01, and

*** indicates *p* < 0.001. Coefficients for race/ethnicity (0 = White, 1 = non-White), sex/gender (0 = cis woman, 1 = non-cis woman), professional status (0 = certified genetic counselors, 1 = genetic counseling trainees), and year in training (0 = 1st year, 1 = 2nd year) were based on Point Biserial correlations. Coefficients for “year in practice” were based on *n* = 107, and those for “year in training” were based on *n* = 108. N/A, Not applicable.

## Data Availability

Data collected for this study and the corresponding codebook are available through the publicly available repository OSF REGISTRIES. The data file is available at https://doi.org/10.17605/OSF.IO/7S5MA.

## What is known about this topic

Distinguishing four types of bias (implicit prejudice, implicit stereotyping, explicit prejudice, and explicit stereotyping) is critical because each type is associated with different clinical outcomes. Prior research has documented moderate implicit racial prejudice toward Black Americans among genetic counselors in North America, but the data come from only two small samples.

## What this paper adds to the topic

This is the first study to assess all four types of bias in a new sample of genetic counselors.
