# Supplementary material for: Implicit and explicit racial prejudice and stereotyping toward Black (vs. White) Americans: The prevalence and variation among genetic counselors in North America
Source: J Genet Couns. Author manuscript; Available in PMC 2023 May 9. (PMC10168592; doi:10.1002/jgc4.1648)
Supplement: Tables S1 and S2 [file NIHMS1891730-supplement-Tables_S1_and_S2.pdf]

Table 5

Means, Standard Deviations, and Range of Each Specific Explicit Stereotype among Genetic Counselors

	M (SD)		*Range*	Certified genetic counselors-genetic counseling trainees comparison		
				t	*p*	*95% CI*
(GENERAL STEREOTYPES)						
Intelligent						
Overall	-0.61**	(1.03)	-5.00 to 2.00			
Certified genetic counselors	-0.58	(0.97)	-5.00 to 1.00			
Genetic counseling trainees	-0.64	(1.09)	-5.00 to 2.00	0.42	.67	[-0.22, 0.34]
Educated						
Overall	-0.17**	(0.93)	-5.00 to 2.00			
Certified genetic counselors	-0.25	(0.92)	-4.00 to 2.00			
Genetic counseling trainees	-0.09	(0.93)	-5.00 to 2.00	-1.26	.21	[-0.41, 0.09]
Industrious						
Overall	-0.25**	(0.81)	-5.00 to 3.00			
Certified genetic counselors	-0.26	(0.69)	-3.00 to 1.00			
Genetic counseling trainees	-0.23	(0.92)	-5.00 to 3.00	-0.27	.79	[-0.25, 0.19]
Responsible						
Overall	-0.60**	(0.92)	-5.00 to 1.00			
Certified genetic counselors	-0.54	(0.93)	-5.00 to 1.00			
Genetic counseling trainees	-0.67	(0.91)	-5.00 to 0.00	0.99	.32	[-0.12, 0.37]
Playful						

Overall	-0.22**	(0.77)	-5.00 to 1.00			
Certified genetic counselors	-0.22	(0.75)	-3.00 to 1.00			
Genetic counseling trainees	-0.23	(0.78)	-5.00 to 1.00	0.16	.88	[-0.19, 0.22]
Humorous						
Overall	-0.39**	(0.77)	-5.00 to 1.00			
Certified genetic counselors	-0.28	(0.67)	-3.00 to 1.00			
Genetic counseling trainees	-0.50	(0.85)	-5.00 to 1.00	2.11	.04	[0.01, 0.43]
Cheerful						
Overall	-0.30**	(0.77)	-5.00 to 2.00			
Certified genetic counselors	-0.18	(0.74)	-3.00 to 2.00			
Genetic counseling trainees	-0.43	(0.88)	-5.00 to 1.00	2.25	.03	[0.03, 0.47]
Athletic						
Overall	-0.23**	(0.74)	-4.00 to 2.00			
Certified genetic counselors	-0.14	(0.65)	-3.00 to 2.00			
Genetic counseling trainees	-0.43	(0.88)	-5.00 to 1.00	1.84	.07	[-0.01, 0.38]
Exploitative						
Overall	1.21**	(1.43)	-2.00 to 5.00			
Certified genetic counselors	1.05	(1.41)	-2.00 to 5.00			
Genetic counseling trainees	1.38	(1.44)	-1.00 to 5.00	-1.71	.09	[-0.72, 0.05]
Stubborn						
Overall	0.73**	(1.11)	-2.00 to 5.00			
Certified genetic counselors	0.61	(1.06)	-2.00 to 5.00			
Genetic counseling trainees	0.84	(1.16)	-2.00 to 5.00	-1.55	.12	[-0.53, 0.06]

Materialistic						
Overall	0.89**	(1.21)	-5.00 to 4.00			
Certified genetic counselors	0.85	(1.11)	-1.00 to 4.00			
Genetic counseling trainees	0.93	(1.32)	-5.00 to 4.00	-0.46	.65	[-0.40, 0.25]
Sheltered						
Overall	1.45**	(1.50)	-2.00 to 5.00			
Certified genetic counselors	1.20	(1.43)	-2.00 to 5.00			
Genetic counseling trainees	1.69	(1.53)	-1.00 to 5.00	-2.47	.01	[-0.90, -0.10]
Poor						
Overall	0.19*	(1.05)	-3.00 to 5.00			
Certified genetic counselors	0.09	(1.13)	-3.00 to 5.00			
Genetic counseling trainees	0.28	(0.96)	-2.00 to 4.00	-1.29	.20	[-0.47, 0.10]
Lazy						
Overall	0.95**	(1.18)	-1.00 to 5.00			
Certified genetic counselors	0.84	(1.15)	-1.00 to 5.00			
Genetic counseling trainees	1.06	(1.20)	-1.00 to 5.00	-1.39	.17	[-0.54, 0.09]
Violent						
Overall	0.91**	(1.30)	-2.00 to 5.00			
Certified genetic counselors	0.90	(1.25)	-2.00 to 5.00			
Genetic counseling trainees	0.93	(1.34)	-2.00 to 5.00	-0.16	.87	[-0.38, 0.32]
Complaining						
Overall	1.11**	(1.25)	-2.00 to 5.00			

Certified genetic counselors	1.01	(1.22)	-2.00 to 5.00			
Genetic counseling trainees	1.20	(1.27)	-1.00 to 5.00	-1.14	.26	[-0.53, 0.14]
(STEREOTYPES SPECIFIC TO MEDICINE)						
Age slowly						
Overall	-0.38**	(1.09)	-4.00 to 3.00			
Certified genetic counselors	-0.33	(1.05)	-4.00 to 3.00			
Genetic counseling trainees	-0.43	(1.12)	-4.00 to 3.00	0.60	.55	[-0.20, 0.38]
Have thick skin						
Overall	-0.15*	(0.82)	-5.00 to 2.00			
Certified genetic counselors	-0.13	(0.79)	-4.00 to 2.00			
Genetic counseling trainees	-0.18	(0.86)	-5.00 to 2.00	0.48	.63	[-0.17, 0.28]
Have sensitive sense of smell						
Overall	0.04	(0.82)	-3.00 to 3.00			
Certified genetic counselors	0.01	(0.82)	-3.00 to 3.00			
Genetic counseling trainees	0.82	(1.62)	-2.00 to 3.00	-0.58	.56	[-0.28, 0.16]
Have strong immune systems						
Overall	-0.36**	(0.93)	-4.00 to 2.00			
Certified genetic counselors	-0.27	(0.98)	-4.00 to 2.00			
Genetic counseling trainees	-0.44	(0.88)	-3.00 to 1.00	1.37	.17	[-0.08, 0.42]
Tolerate pain well						
Overall	-0.01	(0.95)	-3.00 to 4.00			
Certified genetic counselors	-0.07	(0.94)	-3.00 to 4.00			
Genetic counseling trainees	0.06	(0.96)	-3.00 to 3.00	-1.08	.28	[-0.39, 0.12]

Medically compliant						
Overall	-0.29**	(0.80)	-4.00 to 3.00			
Certified genetic counselors	-0.26	(0.79)	-4.00 to 2.00			
Genetic counseling trainees	-0.31	(0.82)	-3.00 to 3.00	0.48	.63	[-0.16, 0.27]
Have high levels of health literacy						
Overall	0.10	(0.98)	-3.00 to 4.00			
Certified genetic counselors	0.05	(1.00)	-3.00 to 3.00			
Genetic counseling trainees	0.16	(0.96)	-3.00 to 4.00	-0.83	.41	[-0.37, 0.15]
Motivated to improve their health						
Overall	-0.23**	(0.83)	-4.00 to 3.00			
Certified genetic counselors	-0.25	(0.86)	-4.00 to 3.00			
Genetic counseling trainees	-0.21	(0.80)	-3.00 to 2.00	-0.35	.73	[-0.26, 0.18]
Adhere to clinical recommendations						
Overall	-0.29**	(0.82)	-3.00 to 2.00			
Certified genetic counselors	-0.25	(0.84)	-3.00 to 2.00			
Genetic counseling trainees	-0.33	(0.81)	-3.00 to 2.00	0.72	.47	[-0.14, 0.30]
Medically cooperative						
Overall	-0.17*	(0.88)	-3.00 to 4.00			
Certified genetic counselors	-0.15	(0.92)	-3.00 to 3.00			
Genetic counseling trainees	-0.22	(0.85)	-2.00 to 4.00	0.60	.55	[-0.16, 0.31]
Mistrustful of the healthcare system						
Overall	-1.08**	(1.32)	-5.00 to 3.00			

Certified genetic counselors	-1.00	(1.14)	-5.00 to 1.00			
Genetic counseling trainees	-1.17	(1.48)	-5.00 to 3.00	0.93	.36	[-0.19, 0.52]
Skeptical of genetic testing						
Overall	-0.34**	(1.09)	-3.00 to 3.00			
Certified genetic counselors	-0.25	(1.07)	-3.00 to 3.00			
Genetic counseling trainees	-0.44	(1.11)	-3.00 to 2.00	1.23	.22	[-0.11, 0.48]
Worried about privacy of genetic test results						
Overall	-0.02	(0.92)	-4.00 to 2.00			
Certified genetic counselors	0.15	(0.80)	-3.00 to 2.00			
Genetic counseling trainees	-0.19	(1.01)	-4.00 to 2.00	2.70	.01	[0.09, 0.58]
Mistrustful of genetic counselors						
Overall	-0.60**	(1.08)	-4.00 to 3.00			
Certified genetic counselors	-0.46	(0.98)	-3.00 to 2.00			
Genetic counseling trainees	-0.73	(1.15)	-4.00 to 3.00	1.88	.06	[-0.01, 0.56]
Don't want to know about future health risks						
Overall	0.23*	(1.06)	-3.00 to 3.00			
Certified genetic counselors	0.22	(1.08)	-3.00 to 3.00			
Genetic counseling trainees	0.24	(1.05)	-3.00 to 3.00	-0.11	.91	[-0.30, 0.27]
Knowledgeable about family health history						
Overall	-0.09	(0.95)	-3.00 to 4.00			
Certified genetic counselors	-0.14	(0.85)	-3.00 to 2.00			
Genetic counseling trainees	-0.05	(1.04)	-3.00 to 4.00	-0.72	.47	[-0.35, 0.16]

Cannot afford genetic counseling services financially						
Overall	0.01	(1.04)	-3.00 to 5.00			
Certified genetic counselors	0.04	(0.97)	-3.00 to 3.00			
Genetic counseling trainees	-.02	(1.11)	-3.00 to 5.00	0.39	.70	[-0.22, 0.34]

Note. * indicates a significant one-sample t-test (the secondary analysis) at $p < .05$ and ** at $p < .01$. *t*-tests presented within the table are independent sample t-tests, comparing certified genetic counselors and genetic counseling trainees.

Table 6

Percentage of Genetic Counselors Endorsing Specific Explicit Stereotypes as More True for Black Americans than White Americans

	More true for Black Americans than for White Americans (scores > 0)	More/less true for neither group (score = 0)	More true for White Americans than for Black Americans (scores < 0)
(GENERAL STEREOTYPES)			
Intelligent	40.0	**57.7**	2.4
Educated	25.6	**59.1**	15.4
Industrious	26.5	**65.1**	8.4
Responsible	42.3	**56.3**	1.4
Playful	22.3	**69.3**	8.4
Humorous	30.7	**67.0**	2.3
Cheerful	30.2	**60.9**	8.9
Athletic	23.3	**70.2**	6.5
Exploitative	5.1	34.9	**60.0**
Stubborn	4.2	**48.8**	47.0
Materialistic	3.7	44.7	**51.6**
Sheltered	3.7	30.2	**66.1**
Poor	14.9	**60.0**	25.1
Lazy	3.3	43.3	**53.4**
Violent	5.1	44.2	**50.7**
Complaining	1.9	40.9	**57.2**

(STEREOTYPES SPECIFIC TO MEDICINE)			
Age slowly	32.1	**56.7**	11.2
Have thick skin	20.9	**67.9**	11.2
Have sensitive sense of smell	17.2	**66.0**	16.7
Have strong immune systems	31.6	**58.6**	9.8
Tolerate pain well	19.5	**59.5**	20.9
Medically compliant	28.4	**65.6**	6.0
Have high levels of health literacy	16.3	**58.1**	25.6
Motivated to improve their health	27.9	**62.3**	9.8
Adhere to clinical recommendations	31.6	**59.1**	9.3
Medically cooperative	28.8	**57.7**	13.5
Mistrustful of the healthcare system	**60.5**	34.4	5.1
Skeptical of genetic testing	36.7	**48.4**	14.9
Worried about privacy of genetic test results	19.1	**59.1**	21.9
Mistrustful of genetic counselors	**47.9**	42.3	9.8
Don’t want to know about future health risks	14.4	**57.7**	27.9
Knowledgeable about family health history	25.6	**57.7**	16.7
Cannot afford genetic counseling services financially	22.8	**54.0**	23.3

Note. The total percentage of some stereotypes does not add to 100 due to rounding. Items under the “General Stereotypes” were categorized based on the stereotype content documented in prior research (Wittenbrink et al., 1997). Specifically, attributes were organized by a combination of racial categories (White vs. Black) and valence (positive vs. negative): (1) positive White stereotypes (intelligent, educated, industrious, responsible); (2) positive Black stereotypes (playful, humorous, cheerful, athletic); (3) negative White stereotypes (exploitative, stubborn, materialistic, sheltered); (4) and negative Black stereotypes (poor, lazy, violent, complaining). Items under the “Stereotypes Specific to Medicine” were also categorized into three groups: (1) stereotypes about

biological differences (the first 5 items); (2) stereotypes about health-related attitudes and beliefs (the next 6 items); and (3) stereotypes specifically in the context of genetic counseling (the last 6 items).
